# Partnering on the PRAISE Program: Putting Health Equity into Practice

**DOI:** 10.1089/heq.2016.0007

**Published:** 2017-01-01

**Authors:** Ameena Batada, JeWana Grier-McEachin, Kathey Avery

**Affiliations:** ^1^ Department of Health and Wellness, University of North Carolina Asheville, Asheville, North Carolina.; ^2^ Asheville Buncombe Institute of Parity Achievement (ABIPA), Asheville, North Carolina.

**Keywords:** faith-based, church, African American, health disparities, interventions, community-based

## Abstract

**Purpose:** The purpose of Preventive Health Education Resulting in Action Inspiring Success for Everyone (PRAISE) was to develop a community-driven program to encourage and support churches in sustainable health promotion and assessment efforts to improve African Americans' health knowledge and behaviors in Asheville, North Carolina.

**Methods:** The PRAISE program provided technical support toward gaining recognition and an award for health promotion activities to 10 churches in year 1 and 5 additional churches in year 2. The Results-Based Accountability^©^ (RBA) framework involved documentation of church health promotion activities and surveys of a convenience sample of congregants at nine churches before (presurvey, *n*=270) and after (postsurvey, *n*=241) the intervention. Differences in frequency of conduct of and participation in church health promotion activities and in congregant health knowledge and behaviors were assessed in 2015 and 2016.

**Results:** Fourteen of the churches engaged in at least one health promotion activity and more than half offered healthier foods at gatherings, offered exercise opportunities, and held at least three health education activities. Seventy-two percent of congregants reported participating in at least one church health activity at postsurvey compared with 58% at presurvey. The proportion of congregants who had personal health knowledge and the proportion that rated their health as good or better were higher at postsurvey.

**Conclusion:** Building on years of trust and collaboration among churches, local community organizations, and an academic partner, PRAISE in its first 2 years suggests that a community-driven approach can support health promotion and healthy behaviors, advancing health equity efforts.

## Introduction

African Americans disproportionately experience health conditions such as cardiovascular diseases and diabetes,^1^ die from health problems such as breast and other specific cancers,^2^ and have less access to and lower quality healthcare.^3^ Given these health disparities, health equity, “the principle underlying a commitment to reducing disparities in health and its determinants,”^4^ is among the priorities of the U.S. Department of Health and Human Services' *Healthy People 2020*^5^ and is the priority of the American Public Health Association.^6^ Furthermore, leaders at the Centers for Disease Control and Prevention (CDC) and other federal agencies have outlined strategies for working toward health equity, which included considering sociodemographic characteristics; taking advantage of the evidence base; leveraging effective multisectoral collaboration; supporting clustering of interventions; engendering meaningful participation by community members; and ensuring rigorous planning and evaluation.^7^

For more than two decades, community-based participatory research (CBPR) interventionists, working with predominantly African American churches, have employed many of these strategies. CBPR in health is “a collaborative approach to research that equitably involves all partners in the research process and recognizes the unique strengths that each brings. CBPR begins with a research topic of importance to the community, with the aim of combining knowledge and action for social change to improve health outcomes and eliminate health disparities.”^8^ CBPR investigators have identified African American churches to be important partners in health promotion because of their historic role as promoter and supporter of spiritual, as well as social, emotional, and physical well-being.^9^ A 2016 review of 16 CBPR studies to improve diet and nutrition in African American church communities found that interventions can improve diet, physical activity, nutrition, and weight management.^10^

According to DeHaven et al.'s review of published literature, faith-placed programs (usually originating as a study) appeared the most often (43.4%), followed by collaborative programs (32.1%), and faith-based (usually church-initiated) programs (24.5%).^11^ Reporting on outcomes follows a similar pattern, with faith-placed programs most likely to report on outcomes (75% of studies), followed by collaborative (37.5%), and faith-based (30.8%) programs. The authors recommended more effectiveness (vs. efficacy) studies and collaborations to measure outcomes. In this vein, Austin and Harris in 2011 discussed the potential of community-based participatory action research (CBPAR) in working with African American churches. In their study to understand the role of the church health ministry, they declared that “the black church may create an empowering environment to address the myriad health issues its members and the African American community confront.”^12^

The myriad health issues, including the social determinants of health and the realities of our country's historical and ongoing challenges of racism, behoove us to go beyond short-term disease-specific interventions. As Ferrer of the W.K. Kellogg Foundation states, “There is an urgent need to lift up authentic narratives that reflect the complexity of lives lived within the construct of systems and practices that for far too long have assigned value to people based on the color of their skin.”^13^ Ferrer emphasizes working from the “…belief that those most affected by a situation or challenge have the inherent capacity to identify and lead the change.”

The belief in the inherent capacity of African American churches and a desire to use community-driven approaches to build a *Culture of Health*^14^ led us to develop the PRAISE (Preventive Health Education Resulting in Action Inspiring Success for Everyone) program. We started as a community organization engaging in health promotion with churches and built into this work an intervention study. The community organization is the Asheville Buncombe Institute of Parity Achievement (ABIPA), an organization that for over 10 years has improved health conditions for African Americans by providing education, health services, and advocacy from a unique understanding of the African American experience and a demonstrated ability to increase collaboration, connection, awareness, and trust across diverse segments of the community^15^ in the Asheville, NC, area. The academic partner is the University of North Carolina Asheville (UNCA), specifically faculty and students in the Department of Health and Wellness. Our purpose in this article is to describe an example of how a community-driven church health promotion program funded by a community grant may measure and share its impact. With individual churches leading their own programs with the support of ABIPA and UNCA, we are putting into practice the principles of health equity.

Our collaboration began with a student group project in a class on community health and, in the next semester, continued with a study of perceptions of health among African Americans. Based on the collected data, we co-developed church-specific interventions at the churches. Of these collaborations, we developed PRAISE to encourage and support churches in health promotion and policy change. The program has been funded at $25,000 each year for 3 years by Mission Health Community Investments and is not affiliated with a previous PRAISE! Project developed by Ammerman et al.^16^

### The PRAISE program

**FIG. 1.**
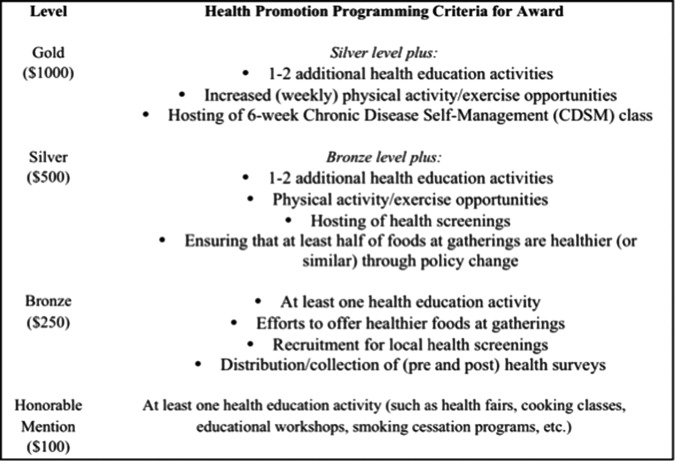
Award levels of the PRAISE program and corresponding criteria for churches to achieve the awards. PRAISE, Preventive Health Education Resulting in Action Inspiring Success for Everyone.

The PRAISE program design emerged out of ABIPA staff's observation that churches evolve through a process of congregational health promotion, which is supported by research on church readiness for change for health promotion.^17^ Based on this process, we developed PRAISE similar to the approach taken by Prevention Partners' recognition programs,^18^ creating criteria for achieving various levels of PRAISE recognition. These levels include Gold, Silver, Bronze, and Honorable Mention, and to achieve each level, churches need to conduct health promotion activities and implement policies (Fig. 1). Although the design of the intervention was organic, it reflects critical elements for faith-based programs, such as paying attention to the partnership, understanding cultural/social contexts, delivery by the community, offering financial incentives, and planning for sustainability.^19^ ABIPA staff offered several evidence-based components to churches, such as *Body & Soul* materials,^20^ *Chronic Disease Self-Management* (*CDSM*)^21^ sessions, and others. The faculty researcher offered guidance and feedback on intervention monitoring and evaluation at the program level. The goal of the PRAISE program was to increase congregants' knowledge of their personal health and their health behaviors through improvements in church health policies and education.

## Methods

The PRAISE program evaluation involved primarily documentation using organizational records of meetings with churches, church agreements, and surveying of church leadership and congregants. Research activities were approved by the Institutional Review Board (IRB) at UNCA.

We utilized the Results-Based Accountability^©^ (RBA) approach, on which both community organization and academic researcher had received training. RBA is part of a growing effort to clarify program evaluation activities such that they are user-friendly for organizations, and its scorecard indicators are useful when collected by multiple community organizations.^22^ The RBA scorecard is part of the community health improvement process (CHIP) of the local Buncombe County Department of Health and Human Services and was preferred by the PRAISE program funder. RBA involves Turning the Curve*—*identifying the areas of improvement—and asks three questions related to the program: (1) What did we do? (2) How well did we do it? (3) Is anybody better off?^22^

Our previous survey data on perceptions of health of African Americans in Asheville provided us with a snapshot to consider how we might be Turning the Curve. To answer the first question, a desk review was conducted. To answer the second question, we tracked the health promotion activities offered at the churches and asked on congregant questionnaires whether they participated in the activities. To answer the third question, we asked congregants to report on their knowledge of their own health status and their health behaviors. The congregant questionnaire included questions about their perception of their general health status; their knowledge of their numbers for height, weight, blood pressure level, and blood cholesterol level; eating, physical activity, and smoking behaviors; church health activities and their participation in them; and demographic information. Several questions were adapted from the Behavioral Risk Factor Surveillance System (BRFSS),^23^ including the question on general health status, which has been shown to be a good reflection of objective health status.^24^

Over nearly 2 years of PRAISE, staff sent letters about the program, offering assistance with health promotion efforts to 50 area churches based on earlier participation in programs with ABIPA and/or because they are part of an association of African American churches. Based on requests, the ABIPA executive director and nurse educator provided in-person technical assistance to 10 churches in year 1 and to 15 (5 additional) churches in year 2. As participating churches joined the program at different time points, congregant presurveys were planned in the first 2 months of program participation and postsurveys were planned at the end of each year of church participation. Churches distributed and collected surveys from all congregants in attendance and returned them to ABIPA once in early 2015 and again in August 2016, and UNCA research assistants entered the data. Descriptive analyses comparing pre- and postsurvey are presented here, although statistical tests of significance between groups were not employed due to the nonequivalent samples.

Overall, 270 congregants completed presurvey questionnaires and 241 from 9 churches completed postsurvey questionnaires ∼1.5 years later. The analysis includes data from churches that returned at least five questionnaires at each time point. The number of returned questionnaires ranged from 5 to 80 from each church. Table 1 presents the sample demographic information at pre- and postsurvey. The sample at postsurvey was a little older, had higher household incomes, and was a little more likely to have health insurance (6% vs. 9%). Across both time points, women, individuals over 44 years, individuals from households with incomes below $50,000 annually, and individuals with health insurance were more likely to complete the questionnaires.

**Table 1.** **Sample Demographic Characteristics at Pre- and Postsurvey**

	Presurvey (*n*=270), *n* %	Postsurvey (*n*=241), *n* %
Gender		
Women	71	70
Men	29	30
Age (years)		
18–29	7	7
30–44	17	14
45–64	51	46
65 and older	25	33
Household income		
<$25,000	43	30
$25,000–$50,0000	35	49
>$500,000	22	21
Insurance status^a^		
No health insurance	9	6
Government	45	45
Employer	41	38
Private	14	17

^a^ The percentage of congregants with each type of health insurance was calculated only among those who reported they had some type of health insurance (*n*=253 at presurvey and *n*=232 at postsurvey).

## Results

### What did we do?

“When did physical health and spiritual health become separated in our churches?”

—ABIPA Nurse Educator

The ABIPA nurse educator often starts classes with the concept of bringing spirit and health back together. Overall, 50 churches were reached with introductory letters and 15 churches with personal communication. The average number of in-person interactions between the executive director and/or the nurse educator and each church was 8. ABIPA also organized four *Know Your Numbers* health screenings at 4 churches, conducted *CDSM* programs at 5 churches, and reoriented churches after changes in leadership at 5 of the churches. ABIPA and UNCA convened a church health conference in April 2015.

### How well did we do it?

“Three John Two states, ‘Beloved, I wish above all things that thou mayest prosper and be in health, even as thy soul prospers.’ The efforts of UNCA and ABIPA as a perfect community collaboration allows our congregations to make this passage literally come to life.”

—Participating Church Pastor

By the end of year 2, 14 of the churches had implemented some policy change to the foods available at their gatherings. Eleven churches had started offering healthy foods as at least half of the options at gatherings. Eleven churches had started regular group exercise opportunities for congregants. Ten churches had provided at least two opportunities for health screenings for their congregants. Eight churches had conducted at least three health education activities, and five had hosted a 6-week *CDSM* program.

From year 1 to year 2, the number of churches achieving Gold status increased from 2 to 5, Silver status from 1 to 2, Bronze status from 3 to 5, and Honorable mentions stayed constant at 2. The attendance at the annual awards ceremony increased from 68 to over 100, demonstrating that health promotion was gaining attention and value. Three church pastors in attendance at the first ceremony committed to coming back to the second ceremony having achieved Gold recognition. Congregants also share their expertise with other churches, such as the juicing lady and the educator on domestic violence issues who now regularly provide workshops for other churches.

The congregant survey provides information about the church's activities. The proportion of congregants reporting that their church was conducting health activities weekly or all the time was higher at postsurvey, including offering healthier foods at gatherings (35% at presurvey and 56% at postsurvey), exercise opportunities (10% and 29%), cooking/nutrition classes (6% and 10%), and other health education activities (21% and 34%). In addition, congregants reported that some health activities were held more than once in a while at postsurvey, including health fairs (10% and 36%) and blood pressure screenings (21% and 29%).

**FIG. 2.**
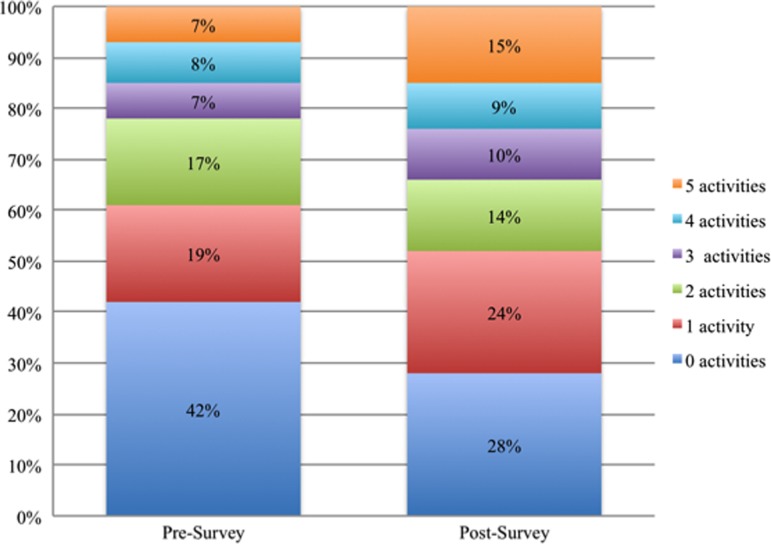
Distribution of congregants according to how many different types of church health promotion activities they engaged in, between 0 and 5, at presurvey and at postsurvey.

The percentage of congregants reporting participation in church health activities also was higher at postsurvey for any church health activity (58% at presurvey and 72% at postsurvey), exercise programs (23% and 34%), and cooking/nutrition classes (20% and 31%). A higher percentage of congregants also reported participating in other health education activities (25% and 46%), health fairs (35% and 41%), and blood pressure screenings (39% and 40%) at postsurvey. The average number of types of church health activities in which congregants participated was higher at postsurvey (1.9) compared with presurvey (1.4). Figure 2 reinforces this finding, displaying the distribution of congregants by the number of different types of church health activities in which they participated.

### Is anybody better off?

“This award means bringing awareness to living a healthy life. It's not being on a diet but changing your lifestyle, making healthy choices, which goes hand in hand with spirituality.”

—Participating Church Congregant

**FIG. 3.**
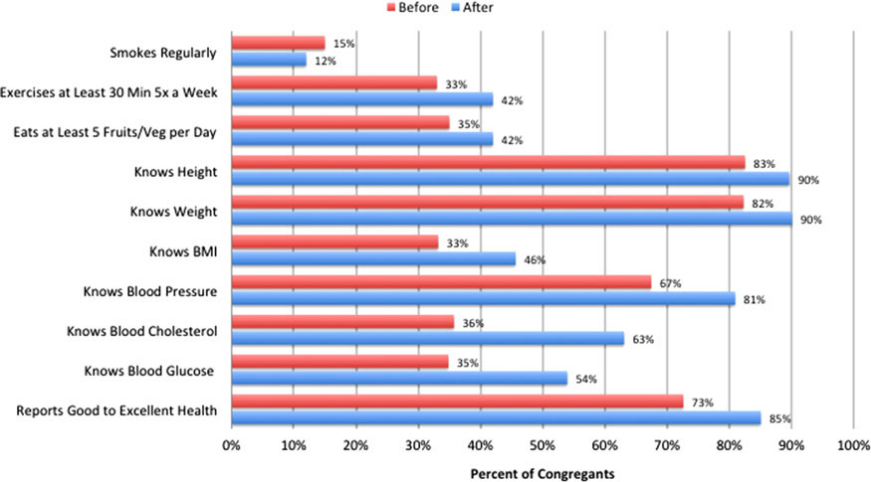
Percentage of congregants who reported that they knew personal health information and percentage of congregants who reported good or better health at presurvey and at postsurvey.

The percentage of congregants who knew their personal health information was higher at postsurvey, including height (83% at presurvey and 90% at postsurvey), weight (82% and 90%), body–mass index (33% and 46%), blood pressure (67% and 81%), blood cholesterol (35% and 63%), and blood glucose (35% and 54%). In addition, a higher proportion (85%) of congregants reported their perceived health status as good, very good, or excellent (as opposed to fair or poor) at postsurvey compared with 73% at presurvey (Fig. 3).

The percentage of congregants eating at least five fruits and nonfried vegetables a day at least 5 days a week was higher at postsurvey (35% at presurvey and 42% at postsurvey) and for congregants exercising 30 min at least 5 days a week (33% and 42%). In addition, the proportion of congregants who reported that they are regular smokers was slightly lower at postsurvey (12%) compared with at presurvey (15%).

## Discussion

The PRAISE program implementation adhered to the planned intervention because it allowed individual churches to request for ABIPA assistance, to obtain ABIPA technical assistance and support on the programming they desired, and to informally learn about and share with other churches. These successes are reflected in the lessons from dissemination research of the American Cancer Society intervention, *Body & Soul*, which recommended assessing church readiness, providing technical assistance, and offering master trainers or coalitions as keys to program success.^25^

Seventy-two percent of congregants reported that they participated in at least one church health activity at postsurvey. In a 2007 study in 11 churches in North Carolina, Odulana et al. found that 30% of congregants participated in church health activities and that they were more likely to participate if they believed that their church played a role in health.^26^ In this program, given that churches included their congregants in the pursuit of a PRAISE award, congregants would have reason to believe that their church was interested in their health. The finding that over half of congregants had participated in a church health activity reflects churches' health promotion activities, likely in conjunction with ABIPA, before the launch of PRAISE.

A higher percentage of congregants also reported knowledge of personal health information and engaged in healthier behaviors at postsurvey. Several other church-based CBPR studies have demonstrated improvements in fruit and vegetable consumption^27,28^ as well as outcomes such as weight or body mass reduction.^29,30^ Most of these studies utilized much more rigorous designs than the current study; however, many were short-term studies involving a disease-specific intervention or curriculum. One project designed with sustainability in mind, the PRAISE! Project developed by Ammerman et al. in North Carolina, was able to meet pastors' expectations around research.^31^

The current research design had several limitations. The questionnaires were distributed according to church schedules and at varying lengths of time between pre- and postsurveying. The convenience sample, which varied in size across churches and time points, is likely not representative of the church demographics. With PRAISE, we anticipated challenges in data collection because the process was new to the churches. All partners did agree that we wanted churches to distribute and collect the questionnaires. The decision to keep researchers out of the churches was intentional because we wanted to maintain the focus on the churches' ownership over all programming. Given the history of experimentation on African Americans by scientific organizations in the United States, we believe this approach is important to our current health equity efforts.

The research also did not involve a control group. As Campbell et al. point out, including a control group poses an ethical dilemma raised in church interventions.^19^ Previous research has used an intention-to-treat design^32^; however, given that funding was primarily for the intervention, we could not afford such a model. Although we are unaware of other major health promotion initiatives, other interventions may have influenced the findings. For example, given that the proportion of respondents with health insurance was higher at postsurvey, it is possible that more congregants had access to care over time. In addition, social desirability bias at postsurvey was likely because congregants were more likely to know about PRAISE goals.

These decisions challenged the standards of rigor in applied research; however, it also was important to cobuild an approach that would be sustainable. As Campbell et al. also state, “Interventions will be most successful if they utilize existing strengths and expertise within the church and build the capacity for churches to be empowered to deliver and sustain interventions over time.”^19^ Now that church leaders are showing increased interest in program data, we hope to strengthen the survey research processes in year 3.

Despite the many research limitations, it is notable that the PRAISE program has health information on more African Americans locally than any other entity, including the county community health assessment conducted in 2012. In addition, because we utilized the RBA framework, our findings are relevant to countywide health improvement process efforts. Furthermore, participating church leaders are interested in the PRAISE results and in utilizing their own church's information for additional programming. The assessments did not measure many of the program outcomes observed by program staff, church leaders, and congregants. As such, we hope to collect qualitative data through interviews and case studies.

## Conclusion

The PRAISE program represents the most recent 2 years of over 10 years of groundwork in addressing African American health disparities by ABIPA and 5 years of partnership with UNCA. To the program collaborators, the program also is an example of how a grant-funded, community-driven, and collaborative health promotion program can measure, present, and utilize outcomes, supporting sustainability by putting health equity into practice.

## Abbreviations Used

ABIPA: Asheville Buncombe Institute of Parity Achievement
BRFSS: Behavioral Risk Factor Surveillance System
CBPAR: community-based participatory action research
CBPR: community-based participatory research
CDC: Centers for Disease Control and Prevention
*CDSM*: *Chronic Disease Self-Management*
CHIP: community health improvement process
IRB: Institutional Review Board
PRAISE: Preventive Health Education Resulting in Action Inspiring Success for Everyone
RBA: Results-Based Accountability^©^
UNCA: University of North Carolina Asheville
